# Suicide prevention through means restriction: Impact of the 2008-2011 pesticide restrictions on suicide in Sri Lanka

**DOI:** 10.1371/journal.pone.0172893

**Published:** 2017-03-06

**Authors:** Duleeka W. Knipe, Shu-Sen Chang, Andrew Dawson, Michael Eddleston, Flemming Konradsen, Chris Metcalfe, David Gunnell

**Affiliations:** 1 South Asian Clinical Toxicology Research Collaboration (SACTRC), Faculty of Medicine, University of Peradeniya, Peradeniya, Sri Lanka; 2 School of Social and Community Medicine, University of Bristol, Canynge Hall, Bristol, United Kingdom; 3 Institute of Health Behaviors and Community Sciences and Department of Public Health, College of Public Health, National Taiwan University, Taipei, Taiwan; 4 Central Clinical School, University Sydney, Sydney, Australia; 5 Pharmacology, Toxicology & Therapeutics, University/BHF Centre for Cardiovascular Science, University of Edinburgh, Edinburgh, United Kingdom; 6 Department of Public Health, Faculty of Health and Medical Sciences, University of Copenhagen, Copenhagen, Denmark; Universitat Wien, AUSTRIA

## Abstract

**Objective:**

To investigate the effect of 3-year phased bans of the pesticides dimethoate and fenthion in 2008–2010, and paraquat in 2009–2011, on suicide mortality in Sri Lanka.

**Methods:**

Age-standardised overall, sex-specific, and method-specific suicide rates were calculated using Sri Lankan police data (1989–2015). Using negative binomial regression models, we estimated the change in the rate and number of suicide deaths in post-ban years (2011–15) compared to those expected based on pre-ban trends (2001–10).

**Findings:**

Overall suicide mortality dropped by 21% between 2011 and 2015, from 18.3 to 14.3 per 100,000. The decline in pesticide suicides during this same period was larger than for overall suicides: from 8.5 to 4.2 per 100,000, a 50% reduction. This was accompanied by a smaller concurrent rise in non-pesticide suicide mortality with a 2% increase (9.9 to 10.1 per 100,000). In 2015, the ratio between the observed and expected pesticide suicide rates was 0.49 (95% confidence interval [CI] 0.40, 0.62), corresponding to an estimated 937 (95% CI 574, 1389) fewer pesticide suicides than expected from pre-ban suicide rates. Findings were similar in sensitivity analyses using 2008 or 2012 as commencement of the post intervention period.

**Conclusion:**

Bans of paraquat, dimethoate and fenthion in Sri Lanka were associated with a reduction in pesticide suicide mortality and in overall suicide mortality despite a small rise in other methods. This study provides further evidence for the effectiveness of pesticide regulation in limiting the availability of highly hazardous pesticides and thereby reducing the number of global suicides.

## Introduction

Over 804,000 suicide deaths are estimated to occur every year; nearly 40% of these are in low and middle income countries (LMIC) in South-East Asia [1]. Roughly 30% of all suicide deaths are due to pesticide poisoning, with a substantial proportion (40%) of pesticide related suicides occurring in South-East Asia [2]. Many small scale farmers keep pesticides in the domestic environment making pesticides easily accessible. In the mid-1990s Sri Lanka had one of the highest suicide rates worldwide (47 per 100,000), with 23,655 deaths over 3 years (1994–96) [3]. Approximately 80% of these suicides were due to pesticide poisoning [3, 4].

Restricting access to lethal means is an effective method of suicide prevention, particularly for impulsive acts with low suicidal intent [5]. Restricting access to toxic pesticides has been suggested as the most effective way to reduce the number of suicides in Asia [6], where most self-poisoning is impulsive [7]. Between 1970 and 1997, the Registrar of Pesticides in Sri Lanka banned the import of a number of the most toxic pesticides [8]. Since the mid-1990s, Sri Lanka experienced dramatic reductions in its suicide rate, with 3772 fewer deaths in 2005 compared to 1995 (44% reduction) [3]. This reduction appears to be driven by the implementation of World Health Organisation (WHO) Class I and endosulphan pesticide bans between 1984 and 1997 [3, 8].

Between 2002–2008 in Sri Lanka, the largest single class (35%) of hospital admitted pesticide self-poisoning cases were organophosphorus insecticides, with dimethoate and fenthion having the highest associated case fatality in this group (21% and 15% respectively) [9]. The pesticide with the highest overall case fatality was paraquat (43%); it accounted for 6% of all admissions.

In 2008, Sri Lanka announced a 3-year phased import ban (2008–2011) of paraquat, dimethoate and fenthion [8]. These pesticides were selected for restriction due to their pivotal role in Sri Lankan pesticide suicides over the previous 5–10 years. For paraquat, the restrictions actually started with a reduction in the concentration of all paraquat formulations to 6.5% in 2008, followed by a 3-year phased import ban (2009–2012).

Evidence from South Korea [10] and Samoa [11] suggests that reduced access to paraquat was associated with a reduction in the national suicide rate. In this analysis we assess the impact on suicide trends of the phased bans of dimethoate, fenthion (2008–2010) and paraquat (2009–2011) in Sri Lanka.

## Methods

### Population data

Censuses were carried out in Sri Lanka in 1981, 2001 and 2011. For the intervening years between 1981 and 2011 we used the adjusted mid-year estimates previously described to ensure that the any changes observed around 2011 would not be driven by the move from estimates to observed data [4]. Mid-year population estimates from 2011 onwards were obtained from the Registrar General for Sri Lanka.

### Suicide data

Suicide data were obtained from the Department of Police, Division of Statistics, Sri Lanka. Age, sex and method-specific data were only available for 1978, 1980, 1982–1985 and 1989–2015. The data are available for selected years online (http://www.police.lk/index.php/crime-trends), and missing years are available upon request directly from the Department of Police. In order to investigate trends, we restricted this analysis to the period with continuous method-specific suicide data available (1989–2015). In 1997 an “insecticide/pesticide poisoning” suicide method category was introduced. Prior to this, pesticide poisoning was categorised in either the “poisoning” or “other method” category. We do not have any further information about the types of methods included in the “other method” category; however we have reason to believe that the majority of these deaths are due to self-poisoning (see supplementary methods–S1 File). Consistent with previous analyses of Sri Lanka’s suicide data [3, 4, 12], we combined the “poisoning” and “other method” categories to represent pesticide poisoning suicide deaths for the years 1989–1996.

### Other related data

Using data available from the Food and Agriculture organisation of the United Nations (http://faostat3.fao.org/) for the years 1992–2013, we assessed changes in the overall use of pesticides in Sri Lanka. Pesticide use is measured as tonnes of active ingredients applied to crops and seeds in the agricultural sector. We also obtained available data on average paddy yield (the main agricultural crop in Sri Lanka) between 1989 and 2014 from the Agriculture and Environment Statistics Division, Sri Lanka (http://www.statistics.gov.lk/agriculture/). We investigated changes in trends of other risk factors for suicide, specifically unemployment and alcohol consumption. Unemployment data were extracted from the Sri Lankan Labour Force Survey for 1990–2015. Available data on per capita alcohol consumption (15+) in litres of pure alcohol (1989–2015) were downloaded from the WHO Global Health Observatory Database (http://www.who.int/gho/database/en/ - accessed 27/09/2016).

### Analysis

In this analysis the outcomes of interest were changes in the incidence of: i) overall (all-method) suicide; and ii) suicides by pesticide ingestion. The exposure was Sri Lanka’s 3-year phased bans of paraquat (2009–2011), dimethoate (2008–2010) and fenthion (2008–2010). Using the 2000 WHO World Standard Population [13] we calculated age-standardised suicide rates among the population aged 8 years and over.

To identify the time period prior to the pesticide bans when trends in Sri Lankan suicide rates were stable, we used Joinpoint regression analysis (http://surveillance.cancer.gov/joinpoint/). We analysed trends in age-standardised pesticide suicide rates and the timing of changes in trends (i.e. “join points”) between 1989 and 2010 (the year of the full enforcement of the first ban). The year 2010 was chosen as the intervention year for the main analysis because by this time the largest contributors to deaths from pesticide self-poisoning (dimethoate and fenthion) were banned. We specified a maximum of 4 join points (the recommended maximum number of join points for the number of observations in our dataset [14]based on the algorithmic recommendations of the software program), and fitted log-linear regression models to detect the number and location of the join points. We presented the results of the best fitting model (S1 Fig and S1 Table), which indicated that 2001 (95% Confidence Interval (CI) 1997–2004) was the start of the period prior to the withdrawal of paraquat, dimethoate and fenthion, with a consistent linear trend in overall suicide rate. The study period used for the main analysis and sensitivity analyses described below was between 1997 (lower limit of the CI) and 2015.

There was statistical evidence of over-dispersion in the Poisson regression models, and therefore we used negative binomial regression to compare suicide rates after the recent pesticide ban in 2011–2015 with those predicted based on pre-ban trends. We calculated rate ratios (and the change in the number of suicides) for each year in 2011–2015 compared to predicted rates based on an extrapolation of trends before the ban (2001–2010). A similar approach has been used previously in investigating the impact of paraquat ban or economic recession on suicide [10, 15]. We fitted negative binomial regression models which included calendar year (single trend term), age group (11 categories starting from 8–16 up to age ≥61) and sex. In order to investigate the effect of the pesticide ban we included a dummy variable for each of the post-ban years (5 dummy variables: 2011–2015). We fitted negative binomial models to the following outcomes:

1. Overall and sex-specific pesticide suicide rates
2. Overall and sex-specific non-pesticide suicide rates
3. Overall and sex-specific overall (all methods) suicide rates

Using the rate ratios and confidence intervals from the negative binomial models we calculated the expected number of suicides in each post-ban year (2011 onwards) by dividing the observed number of suicides by the rate ratio estimate and its confidence interval. The number of deaths prevented was estimated by subtracting the number of expected from the actual suicide deaths. As well as presenting sex stratified results for pesticide and non-pesticide rates, we also formally tested whether the effect of the bans had a differential effect in males versus females by fitting a negative binomial regression model including the post-ban years (2011–2015) as a linear variable and an interaction term of this variable and sex.

In order to test the robustness of our results we: i) changed the start year of the period used to investigate the method-specific suicide trends from 2001 to 1997 or 2004 (the 95% CIs around the join point estimate of 2001); and ii) altered the start of the post-intervention period. We altered the start of post-intervention period from 2010 to: i) 2008 (the start of the phased ban period); and ii) 2012 based on observations that numbers of hospital presentations for paraquat poisoning began to decline most noticeably in 2012 [16] and the fact that the paraquat ban occurred one year after that for dimethoate and fenthion. All negative binomial regression modelling analyses were performed using Stata 14 (StataCorp, College Station, TX).

### Ethics

This study used anonymised publically available data on suicide deaths routinely collected by the Police Department in Sri Lanka.

## Results

**Fig 1:**
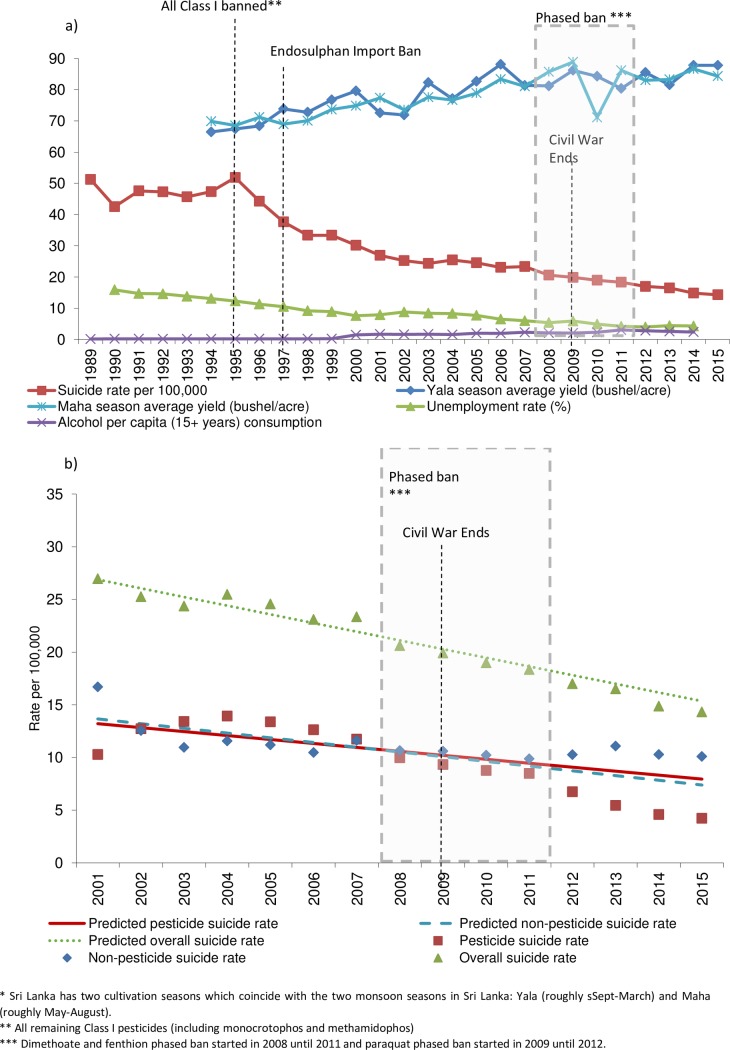
Sri Lanka’s suicide rate (1989–2015). (a) Age-standardised suicide rate, average paddy yield*, unemployment rate and alcohol consumption in Sri Lanka (1989–2015). (b) Age-standardised and predicted pesticide, non-pesticide and overall suicide rate (2001–2015)

Between 2011 (the year when dimethoate and fenthion were fully banned and two years into a three-year paraquat ban) and 2015, the age-standardised overall suicide rate dropped by 21% from 18.3 to 14.3 per 100,000. Pesticides accounted for 38% of suicides deaths in 2000 (start of pre-band period) but dropped to 30% in 2015. The age-standardised pesticide related suicide rate dropped by 50% (from 8.5 to 4.2 per 100,000) between 2011 and 2015 (Fig 1). Conversely, the non-pesticide related suicide rate slightly rose during the same period from 9.9 to 10.1 per 100,000 (a 2% increase). Paddy (rice) crop yields and alcohol consumption increased over the study period, whereas the unemployment rate steadily declined (Fig 1). Available data suggest total pesticide use fluctuated over time (S2 Fig), with the lowest levels of use (insecticides and herbicides combined) observed in 2012. These changes do not appear to be associated with changes in suicide rates.

The pesticide suicide rate for each year post-ban (2011–2015) was lower than that would be expected based on previous trends (2001–2010)–rate ratios (RRs) ranged between 0.90 [95% CI 0.83, 0.97] (2011) and 0.49 [95% CI 0.40, 0.62] (2015), corresponding to an estimated 203 (95% CI 57, 362)937 (95% CI 574, 1389) fewer pesticide suicides than expected in 2011 and 2015 respectively (Table 1). If these RRs were interpreted as percentages, we observed a 10% (95% CI 3%, 17%) reduction in pesticide suicide deaths in 2011 and a 51% (95% CI 38%, 60%) reduction in 2015. There was statistical evidence for a difference from null (RR = 1) for all years. The sex stratified analysis showed a higher absolute reduction in male than female suicides, but there was no statistical evidence for a sex difference in relative changes in pesticide suicide rates (p value for interaction = 0.99) (Table 1). The sum of the estimated changes in the number of male and female pesticide suicides (i.e. the differences between the actual and expected) was not equal to the estimated change in the number of overall (i.e. males and females combined) pesticide suicides in the post-ban years because the sex-specific expected numbers were derived from separate regression models for males and females and would not necessarily add up to the expected number of overall pesticide suicides derived from the regression model for overall pesticide suicide rates.

**Table 1:** Rate ratios and change in the number of suicides in years 2011–2015 after the phased bans of paraquat, dimethoate and fenthion relative to those expected based on trend 2001–10.

	Overall			Male			Female		
	Rate Ratio (95% CI)	P-value	Change in number of suicides (95% CI)	Rate Ratio (95% CI)	P-value	Change in number of suicides (95% CI)	Rate Ratio(95% CI)	P-value	Change in number of suicides (95% CI)
Pesticide suicide rate*									
2011	0.90 (0.83,0.97)	0.01	-203 (-362,-57)	0.90 (0.82,0.98)	0.02	-163 (-308,-31)	0.89 (0.77,1.03)	0.11	-40 (-95,8)
2012	0.72 (0.65,0.80)	<0.01	-538 (-746,-351)	0.72 (0.64,0.81)	<0.01	-445 (-641,-271)	0.81 (0.67,0.99)	0.04	-63 (-137,-2)
2013	0.59 (0.51,0.68)	<0.01	-802 (-1120,-529)	0.60 (0.51,0.72)	<0.01	-629 (-930,-376)	0.60 (0.50,0.71)	<0.01	-132 (-195,-80)
2014	0.52 (0.42,0.64)	<0.01	-909 (-1349,-553)	0.54 (0.44,0.67)	<0.01	-694 (-1064,-396)	0.51 (0.39,0.67)	<0.01	-155 (-255,-79)
2015	0.49 (0.40,0.62)	<0.01	-937 (-1389,-574)	0.52 (0.41,0.66)	<0.01	-714 (-1121,-394)	0.49 (0.39,0.61)	<0.01	-160 (-239,-98)
Non-pesticide suicide rate*									
2011	1.04 (0.95,1.15)	0.40	80 (-113,255)	1.07 (0.96,1.20)	0.19	104 (-55,246)	0.98 (0.88,1.10)	0.78	-9 (-72,48)
2012	1.13 (1.04,1.23)	0.01	239 (74,391)	1.17 (1.06,1.28)	<0.01	229 (94,351)	1.03 (0.91,1.18)	0.62	17 (-54,80)
2013	1.26 (1.16,1.37)	<0.01	471 (312,618)	1.32 (1.20,1.46)	<0.01	426 (291,548)	1.13 (0.99,1.29)	0.07	65 (-5,126)
2014	1.20 (1.09,1.33)	<0.01	368 (179,538)	1.29 (1.15,1.44)	<0.01	373 (219,511)	1.02 (0.85,1.21)	0.85	8 (-86,87)
2015	1.23 (1.10,1.37)	<0.01	394 (188,578)	1.29 (1.15,1.44)	<0.01	362 (216,494)	1.09 (0.90,1.33)	0.38	43 (-59,128)
Overall suicide rate*									
2011	0.97 (0.92,1.02)	0.21	-129 (-339,71)	0.98 (0.93,1.04)	0.49	-59 (-232,106)	0.95 (0.86,1.04)	0.27	-48 (-139,35)
2012	0.93 (0.88,0.98)	0.01	-282 (-491,-85)	0.93 (0.87,0.99)	0.02	-209 (-397,-33)	0.95 (0.85,1.05)	0.31	-46 (-141,40)
2013	0.92 (0.86,0.98)	0.02	-290 (-541,-56)	0.94 (0.87,1.02)	0.15	-171 (-419,57)	0.92 (0.82,1.02)	0.10	-69 (-161,13)
2014	0.86 (0.79,0.94)	<0.01	-504 (-840,-196)	0.89 (0.81,0.99)	0.03	-302 (-596,-36)	0.82 (0.71,0.95)	0.01	-148 (-276,-37)
2015	0.86 (0.79,0.94)	<0.01	-495 (-828,-191)	0.88 (0.80,0.97)	0.01	-332 (-612,-78)	0.85 (0.73,0.99)	0.04	-119 (-250,-7)

*Compared to 2001–10 trend. Results are based on three different models being fitted for pesticide, non-pesticide and overall suicide deaths and therefore results are not comparable between models. In 2010 there were 2914 male suicide deaths (1441 pesticide and 1473 non-pesticide) and 950 female suicide deaths (353 pesticide and 597 non-pesticide)

The non-pesticide suicide rate for the same period (2011–2015) was higher than would be expected based on previous trends (2001–2010). For example, in 2015 the non-pesticide suicide deaths were 43% higher than expected (relative increase based on pre-ban trend), with an estimated 645 (95% CI 481, 794) more deaths (Table 1). Similar to the pesticide-suicide deaths, the absolute increase was more marked in men than women, but with no statistical evidence for a sex modifying effect in relative changes (p value for interaction = 0.98) (Table 1).

The changes in the overall suicide rate were consistent with the method specific changes, with a reduction in the overall suicide rate greater than that would be expected (RR range 0.86–1.05) (Table 1). In 2015, there were 495 (95% CI 191, 828) fewer suicide deaths than expected (Table 1). The sum of the estimated changes in the number of pesticide and non-pesticide suicides was not equal to the estimated change in the number of overall suicides (i.e. pesticide and non-pesticide suicides combined) because the method-specific expected numbers were derived from separate regression models and thus would not necessarily add up to the expected number of overall suicides derived from the regression model for overall suicide rates.

The sensitivity analyses did not alter our overall conclusions. When we used an alternative starting year for the pre-2010 trend (1997 or 2004, based on the 95% CIs for our estimated join point) results were consistent with each other, although the rate ratio was attenuated in the analysis using 2004 (S2 Table). When we altered the start of the post-ban period from 2011 to 2008 (start of the phased ban), a slightly larger reduction was observed in the suicide rate in 2015 (e.g. pesticide suicides: main analysis 0.49 (95% CI 0.40, 0.62) vs. sensitivity analysis: 0.29 (95% CI 0.22, 0.39)) (Table 2). A later start year of 2012 for the post-ban period showed very similar results to the main analysis (Table 2).

**Table 2:** Sensitivity analysis changing the post-intervention period from 2011–2015 to 2008–2015 and 2012–2015.

	Post intervention period—Rate Ratio (95% CI)	
	2008–2015*	2012–2015**
Pesticide suicide rate		
2008	0.77 (0.69, 0.86)	-
2009	0.72 (0.64, 0.80)	-
2010	0.65 (0.57, 0.74)	-
2011	0.63 (0.55, 0.73)	-
2012	0.49 (0.41, 0.58)	0.75 (0.69, 0.82)
2013	0.38 (0.31, 0.47)	0.62 (0.54, 0.71)
2014	0.32 (0.24, 0.42)	0.55 (0.45, 0.66)
2015	0.29 (0.22, 0.39)	0.52 (0.43, 0.64)
Non-pesticide suicide rate		
2008	1.09 (1.00, 1.19)	-
2009	1.16 (1.04, 1.28)	-
2010	1.17 (1.02, 1.33)	-
2011	1.19 (1.0.2, 1.39)	-
2012	1.31 (1.11, 1.54)	1.11 (1.03, 1.20)
2013	1.48 (1.24, 1.78)	1.24 (1.15, 1.33)
2014	1.45 (1.18, 1.77)	1.18 (1.08, 1.29)
2015	1.50 (1.20, 1.87)	1.20 (1.09, 1.33)
Overall suicide rate		
2008	0.92 (0.86, 0.98)	-
2009	0.91 (0.85, 0.97)	-
2010	0.88 (0.82, 0.94)	-
2011	0.87 (0.81, 0.94)	-
2012	0.82 (0.76, 0.90)	0.94 (0.89, 0.98)
2013	0.81 (0.73, 0.89)	0.93 (0.88, 0.99)
2014	0.75 (0.66, 0.84)	0.87 (0.81, 0.95)
2015	0.74 (0.65, 0.83)	0.87 (0.80, 0.95)

* Compared to 2001–2007 trend.

** Compared to 2001–2011 trend.

## Discussion

Our results suggest that the phased bans of paraquat, dimethoate and fenthion in 2008–2011 were followed by a reduction in suicide deaths in Sri Lanka in 2011–2015. There was evidence that the reduction in the overall suicide mortality was driven by a reduction in the number of pesticide related suicide deaths. The reduction in the number of overall suicide deaths as a consequence of the recent pesticide bans may have been stronger if there were not a concurrent rise in non-pesticide suicides, suggesting that some method substitution may have occurred.

Our findings provide further support for the view that restricting access to high toxicity pesticides, through regulation to remove the most highly hazardous pesticides from agricultural practice [17, 18], is effective in reducing both overall and pesticide suicides. This is consistent with previous works from Sri Lanka investigating the impact of earlier pesticide bans on suicide trends, which showed a reduction in suicides following the bans of the most toxic pesticides [3, 4]. Nevertheless, it is possible that the reduction in suicide deaths in Sri Lanka is a consequence of a more general reduction in the use/availability of pesticides in the country. Data available from the Food and Agriculture Organisation of the United Nations (http://faostat3.fao.org/) show a reduction in the use of insecticides and herbicides from 2010, but this downward trend reversed somewhat in 2013 (S2 Fig); this is inconsistent with the observed reduction in pesticide suicide deaths. Another possible explanation for the reduction in the suicide rate in Sri Lanka could be because of a more general improvement to health care provision. Nevertheless, these improvements are unlikely to lead to the step change observed in pesticide suicide deaths. Lastly, the general downward trend in unemployment rates could have contributed to the reduction in suicide rate in Sri Lanka; however, this could not explain the step change in suicide deaths either.

Evidence from Japan [19] and South Korea [10] also indicates that the introduction of paraquat restrictions in these countries has resulted in a reduction in national suicides. Unlike the findings from South Korea, which found limited evidence of method substitution following paraquat restrictions, we found some evidence suggesting a shift of some pesticide suicides to non-pesticide suicides. This rise in non-pesticide suicides was relatively modest, however, and did not greatly counteract the overall reduction in suicide deaths.

Compared to other countries in the region that have similar developmental levels, the downward suicide trend in Sri Lanka is similar to a decline in suicide deaths and in pesticide suicide deaths in China [20–22], but dissimilar to the trend in India where the suicide rates have fluctuated over time (S3 Fig—data from the National Crime Records Bureau (http://ncrb.nic.in/)). The reasons suggested for the decline observed in China include urbanisation (i.e. reduction in the proportion of the population active in farming and therefore exposed to pesticides), improved educational opportunities and higher divorce rates [21]. Previous analyses of the changes in the suicide rates in Sri Lanka suggested that the changes in suicide rates were not driven by divorce (as this has remained stable overtime) [3].

This study provides further evidence that the restriction of access to high toxicity pesticide reduces suicide rates. Other middle income countries with high suicide rates, like India and China, with a similar agricultural sector and pesticide use as Sri Lanka, may consider implementing pesticide legislations to reduce suicide mortality. The WHO estimates that India and China account for 47% (n = 378 805 in 2012) of global suicides [1],and the main method of suicide in the two countries is pesticide poisoning [23, 24]. In 2013, the WHO launched its Mental Health Action Plan 2013–2020, in which they outlined a global target of reducing the suicide rate by 10% by 2020. If India and China can introduce and enforce pesticide legislation for the most toxic pesticides, this reduction in the global suicide rate is achievable.

### Strengths and limitations

One of the strengths of this study is the availability of age, sex and method specific suicide data at the national level. This wealth of data is unusual for a LMIC country. This dataset, however, is not without its limitations. Between the years of 1998–2004 (during the civil war) suicide data from the northern regions of the country (Mullaitivu, Kilinochchi and Mannar, population 1% of national total) were excluded from the national suicide statistics. A sensitivity analysis including only the years with full island coverage (2004 onwards) did not impact on the overall findings from this study (S2 Table). A further limitation is that we may not be capturing the true incidence of suicide. There are no studies which have investigated the quality or reliability of Sri Lanka’s suicide data. Anecdotal evidence from our field research indicates that the quality of suicide data may vary by police departments, but we have no evidence to suggest that the data quality has changed over time [4].

Time series analyses of data from a single country, as we have here, must be interpreted with caution. There are trends over time in many things, and similar trends in two measures provide no convincing evidence of one causing the other, or of a common underlying cause. A change in a trend coinciding with the point in time at which an event occurs (e.g. a pesticide ban) may lead to the hypothesis that the two are associated [25], but even in this situation the evidence would be more convincing if the same association being observed in multiple situations [26].

## Conclusion

In conclusion, our results suggest that the introduction of pesticide restrictions banning the agricultural use of paraquat, dimethoate and fenthion was followed by a reduction in overall and pesticide-specific suicide mortality rates in Sri Lanka. The largest decrease was observed in men. Whilst we observed some evidence of method substitution, the rise in suicide by other methods was much smaller than the fall in the number of pesticide deaths, resulting in an overall 21% absolute decline in all suicides over five years in 2011–2015. It is, however, important to monitor any rise in non-pesticide suicides and the longer term effect of pesticide bans. The current study provides further evidence to support means restrictions through legislationas an effective method of reducing the overall suicide mortality, particularly as pesticide poisoning is one of the main methods of suicide worldwide.

## Supporting information

S1 File Supplementary methods. (DOCX)

S1 Fig Best fitting model joinpoints model for the period 1989–2010. (DOCX)

S2 Fig Sri Lanka pesticide imports and insecticide/herbicide use from the Food and Agriculture organisation of the United Nations (1999–2013). (DOCX)

S3 Fig Crude suicide rate from the National Crime Bureau for India (1999–2014). (DOCX)

S1 Table Joinpoint regression analysis: annual percent change (APC) and joinpoints (JP) for trends in age-standardised rate of pesticide suicides in Sri Lanka, 1989–2010. (DOCX)

S2 Table Sensitivity analyses: rate ratios in years 2011–2015 after the phased bans of paraquat, dimethoate and fenthion relative to those expected based on trends 1997–2010 or 2004–2010. (DOCX)
