# Correction: Suicide prevention through means restriction: Impact of the 2008-2011 pesticide restrictions on suicide in Sri Lanka

**DOI:** 10.1371/journal.pone.0176750

**Published:** 2017-04-25

**Authors:** Duleeka W. Knipe, Shu-Sen Chang, Andrew Dawson, Michael Eddleston, Flemming Konradsen, Chris Metcalfe, David Gunnell

The following information is missing from the Funding section: DK is an Economic and Social Research Council (UK) postdoctoral fellow (ES/P009735/1).
