# Supplementary material for: Suicide prevention through means restriction: Impact of the 2008-2011 pesticide restrictions on suicide in Sri Lanka
Source: PLoS One. 2017 Mar 6;12(3):e0172893. doi: 10.1371/journal.pone.0172893 (PMC5338785; doi:10.1371/journal.pone.0172893)
Supplement: S1 File — (DOCX) [file pone.0172893.s001.docx]

**S1 File - Supplementary materials**

**Supplementary methods – pesticide suicide data**

Prior to 1997 the method-specific suicide statistics produced by the police in Sri Lanka did not include a separate insecticide/pesticide poisoning category and a large proportion of suicide deaths were coded under an “other methods” category. A full list of the categories are provided in the table below. Consistent with our previous analyses we have combined the “poisoning” and “other methods” categories to represent pesticide suicide death prior to 1997 (1, 2). Whilst we do not have information about the types of suicide deaths which would have been classified as “other methods” we believe that the majority of these will be due to self-poisoning. There are several reasons for this: (i) when looking at the percentage of suicides by each method, in 1982 and 1983, there was over a 2/3 reduction in suicides by “other methods”, with a compensatory rise in self-poisoning suicides but no change in other methods of suicide; ii) in 1997, when the insecticide/pesticide poisoning category was introduced, the percentage of suicides coded as “other methods” dropped from 52% in 1996 to 32% in 1997, whilst suicide by self-poisoning rose from 27% in 1996 to 44% in 1997. Suicide by any other method remained the same, with only a small increase in the percentage of hanging suicides.

**Supplementary methods table** – Categories of suicide deaths coded during different periods

|  | Coding periods | | |
| --- | --- | --- | --- |
|  | 1975-1996 | 1997-2001 | 2002-2010 |
| Suicide methods/categories | Poisoning |  |  |
|  | Hanging | Hanging | Hanging |
|  | Jumping in front of train | Jumping in front of train | Jumping in front of train |
|  | Drowning | Drowning | Drowning |
|  | Burning | Burning | Burning |
|  | Shooting | Shooting | Shooting |
|  | With sharp cutting instrument | With sharp cutting instrument | With sharp cutting instrument |
|  | Other means | Other means | Other means |
|  |  | Insecticide/Pesticide poisoning | Insecticide/Pesticide poisoning |
|  |  | Acetic Acid Poisoning |  |
|  |  |  | Firearms |
|  |  |  | Explosives |
|  |  |  | Ingestion of acids |
|  |  |  | Medicinal drug overdose |
|  |  |  | Plant Poisoning |
|  |  |  | Jumping from a height |
|  |  |  | Ingestion/injection of addictive drugs |

1. Gunnell D, Fernando R, Hewagama M, Priyangika WD, Konradsen F, Eddleston M. The impact of pesticide regulations on suicide in Sri Lanka. Int J Epidemiol. 2007;36(6):1235-42.

2. Knipe DW, Metcalfe C, Fernando R, Pearson M, Konradsen F, Eddleston M, et al. Suicide in Sri Lanka 1975-2012: age, period and cohort analysis of police and hospital data. BMC Public Health. 2014;14(1):839.
