# Supplementary figures and images for: Suicide prevention through means restriction: Impact of the 2008-2011 pesticide restrictions on suicide in Sri Lanka
Source: PLoS One. 2017 Mar 6;12(3):e0172893. doi: 10.1371/journal.pone.0172893 (PMC5338785; doi:10.1371/journal.pone.0172893)

**Supplementary figure 1 –** Best fitting model joinpoints model for the period 1989-2010


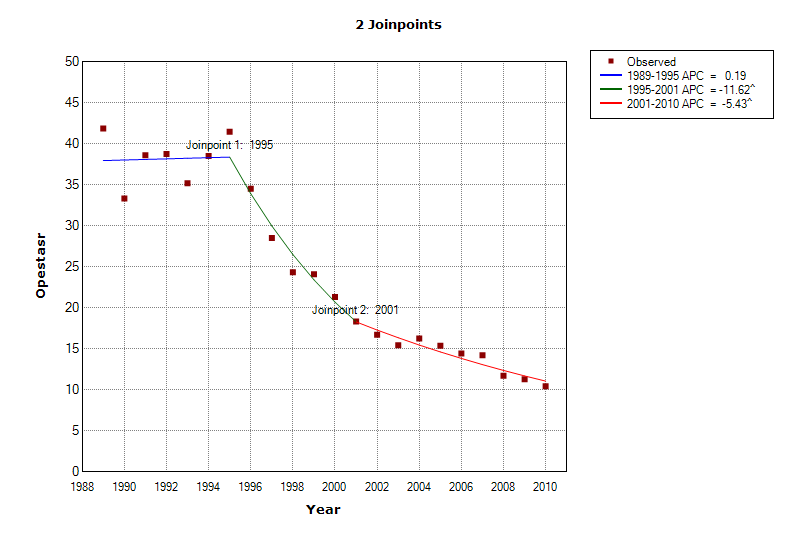

Supplement: S1 Fig — (DOCX) [file pone.0172893.s002.docx]
