# Supplementary material for: Suicide prevention through means restriction: Impact of the 2008-2011 pesticide restrictions on suicide in Sri Lanka
Source: PLoS One. 2017 Mar 6;12(3):e0172893. doi: 10.1371/journal.pone.0172893 (PMC5338785; doi:10.1371/journal.pone.0172893)
Supplement: S1 Table — (DOCX) [file pone.0172893.s005.docx]

**Supplementary table 1** – Joinpoint regression analysis: annual percent change (APC) and joinpoints (JP) for trends in age-standardised rate of pesticide suicides in Sri Lanka, 1989-2010

|  | Segment 1  (1989-1995) | JP1  (95% CI) | Segment 2  (1995-2001) | JPC 2  (95% CI) | Segment 3  (2001-2010) |
| --- | --- | --- | --- | --- | --- |
|  | APC  (95% CI) |  | APC  (95% CI) |  | APC  (95% CI) |
| Pesticide suicides | 0.2  (-2.8, 3.3) | 1995 (1993,1997) | -11.6  (-15.5, -7.5) | 2001 (1997,2004) | -5.4  (-7.7, -3.1) |
