# Supplementary material for: Suicide prevention through means restriction: Impact of the 2008-2011 pesticide restrictions on suicide in Sri Lanka
Source: PLoS One. 2017 Mar 6;12(3):e0172893. doi: 10.1371/journal.pone.0172893 (PMC5338785; doi:10.1371/journal.pone.0172893)
Supplement: S2 Table — (DOCX) [file pone.0172893.s006.docx]

**Supplementary table 2** – Sensitivity analyses: rate ratios in years 2011-2015 after the phased bans of paraquat, dimethoate and fenthion relative to those expected based on trends 1997-2010 or 2004-2010

|  |  | Beginning of study period - Rate Ratio (95% CI) | |
| --- | --- | --- | --- |
|  |  | 1997* | 2004** |
| Pesticide suicide rate | | |  |
|  | 2011 | 0.88 (0.82,0.96) | 1.06 (0.99,1.13) |
|  | 2012 | 0.71 (0.64,0.79) | 0.90 (0.82,0.98) |
|  | 2013 | 0.58 (0.49,0.68) | 0.77 (0.67,0.88) |
|  | 2014 | 0.51 (0.41,0.63) | 0.71 (0.58,0.86) |
|  | 2015 | 0.48 (0.38,0.60) | 0.71 (0.58,0.87) |
| Non-pesticide suicide rate | | |  |
|  | 2011 | 1.17 (1.04,1.31) | 0.96 (0.89,1.04) |
|  | 2012 | 1.29 (1.17,1.43) | 1.02 (0.95,1.10) |
|  | 2013 | 1.47 (1.35,1.61) | 1.12 (1.04,1.21) |
|  | 2014 | 1.45 (1.32,1.59) | 1.05 (0.96,1.16) |
|  | 2015 | 1.51 (1.36,1.68) | 1.05 (0.95,1.17) |
| Overall suicide rate | | |  |
|  | 2011 | 1.02 (0.97,1.08) | 1.01 (0.96,1.06) |
|  | 2012 | 0.99 (0.94,1.05) | 0.98 (0.93,1.04) |
|  | 2013 | 1.00 (0.94,1.06) | 0.99 (0.92,1.06) |
|  | 2014 | 0.94 (0.87,1.02) | 0.94 (0.85,1.03) |

* Compared to 1997-2010 trend

** Compared to 2004-2010 trend
